# Safety and Efficacy of Intratympanic Alpha-Lipoic Acid Injection in a Mouse Model of Noise-Induced Hearing Loss

**DOI:** 10.3390/antiox11081423

**Published:** 2022-07-22

**Authors:** Jae Sang Han, Ye Lin Kim, Hyo Jeong Yu, Jung Mee Park, Yeonji Kim, So Young Park, Shi Nae Park

**Affiliations:** 1 Department of Otorhinolaryngology-Head and Neck Surgery, Seoul St. Mary’s Hospital, College of Medicine, The Catholic University of Korea, 222, Banpo-daero, Seocho-gu, Seoul 06591, Korea; gooddoc@catholic.ac.kr (J.S.H.); yeonji89@gmail.com (Y.K.); 2 Department of Biomedicine & Health Sciences, College of Medicine, The Catholic University of Korea, 222, Banpo-daero, Seocho-gu, Seoul 06591, Korea; kyl1165@hanmail.net (Y.L.K.); yyllzzll@naver.com (H.J.Y.); 3 Department of Otorhinolaryngology-Head and Neck Surgery, Gangneung Asan Hospital, College of Medicine University of Ulsan, 38 Bangdong-gil, Sacheon-myeon, Gangneung-si 25440, Korea; jmpentdoctor@gmail.com; 4 Department of Otorhinolaryngology-Head and Neck Surgery, Yeouido St. Mary’s Hospital, College of Medicine, The Catholic University of Korea, 10, 63-ro, Yeongdeungpo-gu, Seoul 07345, Korea; sypak@catholic.ac.kr

**Keywords:** alpha-lipoic acid, oxidative stress, hearing loss, intratympanic injection

## Abstract

Alpha-lipoic acid (ALA) is an antioxidant with oto-protective effects. In the present study, the safety and effectiveness of ALA therapy after noise-induced hearing loss was confirmed based on the administration method. The safety of intratympanic ALA (IT-ALA) was evaluated with oto-endoscopy and middle ear mucosa morphologic study. Perilymph ALA concentrations according to the administration routes were compared, and the efficacy of ALA was investigated through hearing tests and cochlear histological studies. The middle ear mucosa was swollen 1 week after IT-ALA but completely recovered within 3 weeks. ALA concentration in the perilymph was significantly higher in the IT-ALA group. Recovery of organ of Corti morphology and hearing levels were predominant in the IT-ALA group compared with the intraperitoneal injection group (IP-ALA) and showed similar rescue effects in the IT-dexamethasone group (IT-DEX). Interleukin-1 beta and nuclear factor-kappa B expression was significantly downregulated in the IT-ALA group. IT-ALA showed better cochlear recovery from acoustic trauma with higher inner ear penetration rate than IP-ALA. The rescue effect of IT-ALA after noise-induced hearing loss was similar to IT-DEX; however, the ALA and DEX mechanisms are different. IT-ALA appears to be another safe and effective treatment modality after acoustic trauma and comparable to IT-DEX.

## 1. Introduction

Oxidative stress is defined as an imbalance between the production of reactive oxygen species (ROS) and the defensive mechanism of antioxidants in cells and tissues [1]. Excessive accumulation of ROS adversely affects human cells and oxidative stress is considered responsible for several chronic and degenerative diseases, the aging process, and the acute phase of tissue injury [2]. The organ of Corti (OC) can also be affected by oxidative stress. The role of ROS in ototoxicity, noise-induced hearing loss (NIHL), idiopathic sudden sensorineural hearing loss (ISSNHL), and presbycusis has been reported [3,4]. To reduce the cochlear damage caused by oxidative stress, various studies on antioxidant molecules have been conducted in vitro and in vivo [3,5].

Alpha-lipoic acid (ALA), also known as thioctic acid, protects cells and tissues from oxidative stress through a metal-chelating effect. ROS scavengers regenerate antioxidants and repair oxidative injury [6]. ALA has been reported to have beneficial effects, such as prevention of diabetic neuropathy, protective effects on ischemic injury to the heart and kidneys, and reduction of neurodegeneration [7,8,9]. In addition, the antioxidative effects of ALA on the cochlea have been demonstrated in several animal studies. Systemic administration of ALA showed protective and therapeutic effects on hearing in the cisplatin-induced ototoxicity model [10]. The protective effect of ALA in a kanamycin-induced ototoxicity model and age-related hearing loss model [11,12], and better hearing preservation of ALA after cochlear implantations, have also been reported [13]. However, most of the previous studies were performed in animals using a systemic administration of ALA [14,15,16]. Compared with systemic administration, intratympanic (IT) injection of a drug has several advantages: no or fewer systemic side effects of drugs, higher target organ (cochlea) concentrations, and relatively easy application [17].

Additionally, most animal studies investigating the effect of ALA on the cochlea have used drug-induced hearing loss models [10,11,14,15]. Acoustic trauma triggers inflammatory reactions, oxidative stress, and insufficient blood supply to the inner ear, leading to apoptosis of sensory cells [18]. Several antioxidants, such as glutathione and N-acetylcisteine, have been studied to target oxidative stress, and have been reported to have an oto-protective effect against NIHL [18,19,20]. However, there have been limited studies reporting the effects of ALA on NIHL models [21,22].

Therefore, in the present study, the safety of IT injection of ALA (IT-ALA) was evaluated, and the therapeutic effects compared with IT injection of dexamethasone (IT-DEX) after acoustic trauma based on administration routes. 

## 2. Materials and Methods

### 2.1. Experimental Design

**Figure 1:**
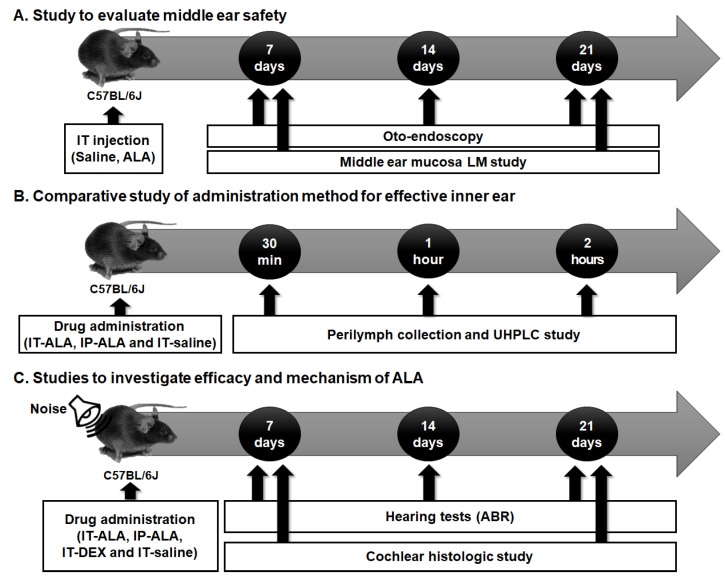
The experimental design scheme. (**A**) The middle ear safety after IT drug administration was investigated using otoendoscopy and middle ear mucosal studies. (**B**) The concentration of ALA in the sampled perilymph was measured according to the administration methods. (**C**) The therapeutic effect of ALA was assessed using histologic studies and hearing tests in the mouse model of NIHL. IT, intratympanic; ALA, alpha-lipoic acid; LM, light microscopic; UHPCL, ultra-high performance liquid chromatography; ABR, auditory brainstem response; NIHL, noise-induced hearing loss.

The experimental protocol is presented in Figure 1. Firstly, the tympanic membranes of the mice were observed with otoendoscopy and middle ear mucosa using light microscopy to investigate the safety of IT-ALA (Figure 1A). Secondly, the ALA concentration in the perilymph was measured using ultra-high performance liquid chromatography (UHPLC) in IT-ALA and intraperitoneal-ALA injection (IP-ALA) groups to compare the pharmacokinetics based on the administration methods (Figure 1B). Lastly, comparison studies were performed using serial hearing tests and cochlear histomorphologic tests to evaluate the efficacy of IT-ALA and investigate the rescue mechanism of ALA after acoustic trauma (Figure 1C). 

### 2.2. Animal Preparation 

All experiments were approved and conducted in accordance with national ethical guidelines and relevant laws. The study protocol complied with the dictates of the Animal Care and Use Committee of the Catholic University of Korea (IACUC-CUMC-2020-0241-02). 

Male C57BL/6J (B6) mice (6 weeks of age; weight 25–30 g) were purchased from the Central Experimental Animal Center (Orient Bio, Sungnam, Korea) and kept in the animal colony of the Catholic University of Korea before experimental procedures began. 

Auditory brainstem response (ABR) and distortion product otoacoustic emission (DPOAE) tests were performed on all mice before noise exposure. In the mouse model of NIHL, 110 dB white noise was administered for 60 min in a specially designed acrylic box (53 cm × 35 cm × 53 cm × 2 cm) with eight compartments placed in a pie-shaped wire-mesh cage; a single mouse in each compartment was exposed to noise. White noise of 10 kHz was generated using random noise generators (B&K Type 1027) placed on top of the box [23]. 

### 2.3. Drug Administration

The animals were divided into the following 4 groups: IT-saline, IT-DEX, IT-ALA, and IP-ALA. The mice in the IT-saline group were administered 0.9% sodium chloride (Dai Han Pharm. Co., Ltd., Seoul, South Korea). The mice in the IT-DEX group were administered a 5 mg/mL concentration of dexamethasone-21-phosphate (Sigma-Aldrich, St. Louis, MO, USA) [18]. In the IP-ALA group, mice were administered 25 mg/kg of ALA for 4 consecutive days [10]. In the IT-ALA group, mice were administered 0.75 mg sodium thioctate (#2319-84-8, Santa Cruz Biotechnology, Santa Cruz, CA, USA) diluted in 10 µL of 1x phosphate-buffered saline (PBS). The pars tensa of the tympanic membranes was punctured using a 30-gauge spiral needle and the drug gently injected ensuring the middle ear cavity was filled. The mice were placed in the decubitus position with the injected ear upward for 15 min to ensure even distribution within the middle ear. Because a tiny perforation remained in the eardrum after the first IT injection, the second and subsequent injections were injected through the perforation to avoid creating multiple holes. All drugs were administered a total of 4 times for 4 consecutive days.

### 2.4. Otoendoscopic Examination

Tympanic membranes were examined before noise exposure and animals were excluded from the study if abnormal findings were observed. The healing process of the tympanic membranes was observed on days 7, 14, and 21 after the 4th IT injection. A rigid 0° otoendoscope (outer diameter, 2.7 mm; length, 3 cm) with Medstar Sharima HGL-100 DC 15 V/150 W (Halogen) light source was used for the examination. 

### 2.5. Perilymph Collection and ALA Concentration Measurement

While the mouse was under anesthesia, a retro-auricular incision was made, and the lateral semi-circular canal was penetrated under a microscope. Perilymph was collected in capillary tubes (Sigma-Aldrich, St. Louis, MO, USA). The collected perilymph samples and standards were diluted and analyzed using an LC–MS/MS equipped with an Agilent 1290 Infinity II LC system (Agilent Technologies, Santa Clara, CA, USA) and a QTRAP^®^ 6500 LC–MS/MS System (SCIEX, Framingham, MA, USA). For liquid chromatographic separation, an Agilent Eclipse XDB–C18 (75 mm × 2.1 mm, 2.7 µm, Agilent Technologies, Santa Clara, CA, USA) was applied with a column temperature of 40 °C. The mobile phase consisted of solvent A (0.1% formic acid in deionized water) and solvent B (0.1% formic acid in acetonitrile) at a flow rate of 0.4 mL/min. The auto-sampler was maintained at 40 °C with an injection volume of 5 µL for all samples. For mass spectrometry separation, a QTRAP^®^ 6500 LC–MS/MS System equipped with electrospray ionization (Turbo VTM ion source) was used. Data acquisition was performed in positive multiple reaction monitoring (MRM) mode with a duration of 13 min and a total cycle of 2516. The ion source potential and collision energy were optimized for each MRM transition. The collision energy was 12 V, and the cell exit potential was 15 V. Nitrogen was used as the curtain gas (35 psi), ion source gas 1 (nebulizer gas, 50 psi), and ion source gas 2 (turbo gas, 50 psi). The ion source temperature was 500 °C, and the ion source voltage was 4500 V.

### 2.6. Light Microscopy

#### 2.6.1. Morphometry of the OC

The morphology of the basal, middle, and apical turns was evaluated using light microscopy of six randomly selected sections from the mid-modiolar area of each turn. The extent of OC degeneration was graded from 1 to 5 in whole numbers using a modification of the rank order method of Leake [24]. The detailed method was previously described [25].

#### 2.6.2. Morphometry of Middle Ear Mucosa

Specimens of the middle ear, including the promontory, bulla, tympanic membrane, and Eustachian tubal orifice, were embedded in paraffin following a dehydration procedure and sectioned for light microscopy (0.5 µm). Periodic acid–Schiff (PAS) staining was performed for morphologic examination. The thicknesses of the TM, mucosa, and submucosa in the inferior tympanum containing the ciliated tract were measured using the modified method of Tsuboi [26]. The thickness of TM, mucosa, and submucosa were measured using the Panoramic viewer software program (3DHISTECH Ltd., Budapest, Hungary).

### 2.7. Hearing Test

Mice were anesthetized with an IP injection of a mixture of zolazepam with tiletamine (35 mg/kg) and xylazine (10 mg/kg). ABRs were recorded using an Intelligent Hearing System (IHS) Smart EP fitted with high-frequency transducers (HFT9911-20-0035) and running high-frequency software version 2.33 (IHS, Miami, FL, USA). Subdermal needle electrodes placed in the vertex (active) and below the left pinna (reference) were connected to a preamplifier filtering from 0.1 to 3 kHz. The acoustic stimuli used comprised a click (100 µs in duration; 31 Hz) and tone bursts of 8, 16, and 32 kHz (1562 µs in duration; cos2-shaped; 21 Hz) that were presented through an inserted earphone and attenuated in 5- to 10-dB steps to determine the thresholds. Evoked potentials were amplified (×200,000), bandpass-filtered (100–3000 Hz), and averaged over 1024 sweeps. Thresholds were determined for the broadband click and for the 8, 16, and 32 kHz tone bursts to determine the lowest level at which distinct ABR wave patterns were recognizable by two investigators.

### 2.8. Immunofluorescence Study

Immunofluorescence assays were performed with two inflammatory markers associated with noise-induced cochlear inflammation and oto-protective mechanism of ALA: interleukin-1 beta (IL-1ß) and nuclear factor-kappa B (NF-κB) [27,28,29]. Animals were sacrificed at 1 week to investigate IL-1ß and NF-κB expression which was reportedly increased at this time point after acoustic trauma [29]. In addition, to investigate the long-term efficacy of IT-ALA after acoustic trauma, the expression of these inflammatory markers was evaluated 3 weeks after drug administration.

The dissected cochlea was immediately cryo-embedded and then frozen in liquid nitrogen. The specimen was cryosectioned at 10 μm in the coronal plane at −20 °C in a Leica CM1850UV Cryostat microtome (Leica Microsystems, Wetzlar, Germany), and stored at −20 °C. The sections were fixed in acetone for 10 min at −20 °C. After washing, they were blocked in 2% BSA for 1 h at room temperature in a dark and humidified chamber, and then were incubated at 4 °C overnight with the IL-1β (#AF-401-NA, R&D Systems, MN, USA) antibody (1:400) and NF-kB (#8242, Cell Signaling Technology, MA, USA) antibody (1:400) in 0.5% BSA. After washing, sections were incubated for 2 h at room temperature with a green fluorescent donkey anti-goat IgG (#ab150129, Abcam, Cambridge, UK) antibody (1:1000) and a red fluorescent goat anti-rabbit IgG (#A21428, Thermo Fisher Scientific Inc., Cleveland, OH, USA) antibody (1:1000). Slides were viewed and photographed by confocal laser scanning microscopy (LSM 900 w/Airyscan, Carl Zeiss, Jena, Germany). Four series of Z-stack images were acquired per muscles at 0.5 μm intervals. A single digital image was reconstructed from the Z stacks using ZEN 2012 software (Carl Zeiss, Jena, Germany).

### 2.9. Data Analysis and Statistics 

All data were statistically analyzed using SPSS 24.0 software (IBM Corp., Armonk, NY, USA). The parametric or nonparametric test was used after the Shapiro–Wilk test for normality. Perilymph concentrations of ALA based on administration method were compared using the Mann–Whitney test. Middle ear mucosal thickness, OC grading scores, and immunofluorescence results were statistically analyzed using the Kruskal–Wallis test and all pairwise comparisons were evaluated using the Mann–Whitney test with Bonferroni correction. All paired data were analyzed using the Wilcoxon signed rank test. The ABR results from the IT-ALA group were compared with the other 3 groups using the Mann–Whitney test with Bonferroni correction. All data are presented as mean ± standard deviation (SD) and *p*-value < 0.05 was considered statistically significant.

## 3. Results

### 3.1. Assessment of the Middle Ear Safety after IT-ALA Administration

**Figure 2:**
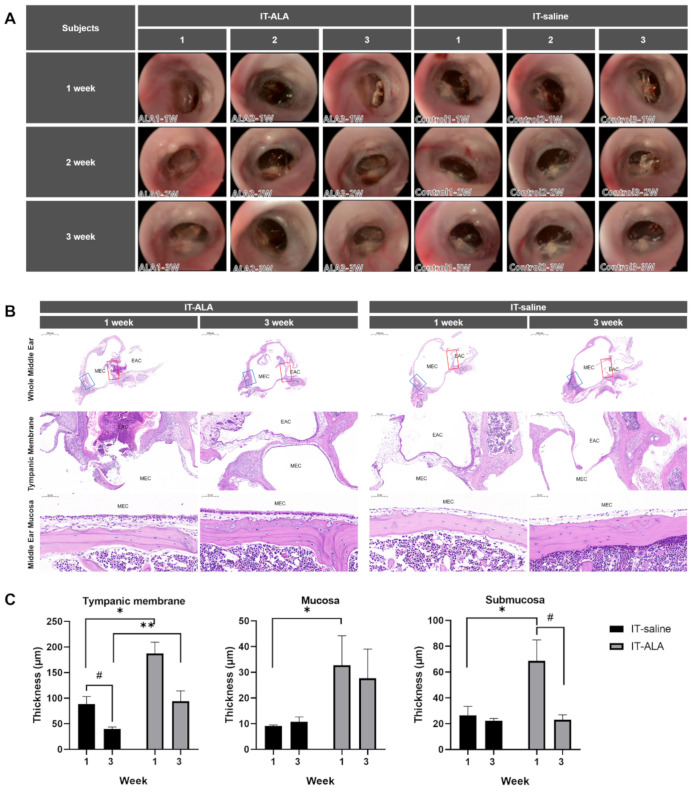
Assessment of the middle ear safety. (**A**) The otoendoscopic findings of the tympanic membrane showed no significant differences between the IT-saline and IT-ALA groups. Representative light microscopic findings (**B**) and thickness analysis of the tympanic membrane, mucosal, and submucosal layers (**C**). A significant increase in the thickness of middle ear mucosa was observed in the IT-ALA group 1 week after injection (*p* < 0.05); however, the difference in mucosa and submucosa thickness between IT-ALA and IT-saline groups decreased 3 weeks after injection except for the tympanic membrane thickness (*p* = 0.01). IT, intratympanic; ALA, α-lipoic acid; DEX, dexamethasone. The red square indicates the tympanic membrane, and the blue square indicates middle ear mucosa posterior to the Eustachian tube. Mann–Whitney test for inter-group comparison, * *p* < 0.05, ** *p* < 0.01, Mann–Whitney test for within-group comparison, # < 0.05.

The serial oto-endoscopic findings obtained at one, two, and three weeks after the IT injections showed no significant differences between IT-saline and IT-ALA groups (Figure 2A). Light microscopic evaluations for the middle ear were conducted one and three weeks after IT-ALA administration. Tympanic membrane as well as mucosal and submucosal layers of the middle ear one week after injections were significantly thicker in the IT-ALA group than in the IT-saline group (*p* < 0.05). However, the thickness of the middle ear mucosa in the IT-ALA group decreased three weeks after injection and the difference in thickness between the two groups was observed only in the tympanic membrane (*p* = 0.01; Figure 2B,C).

### 3.2. Perilymph Concentration of ALA Based on Drug Administration Methods

**Figure 3:**
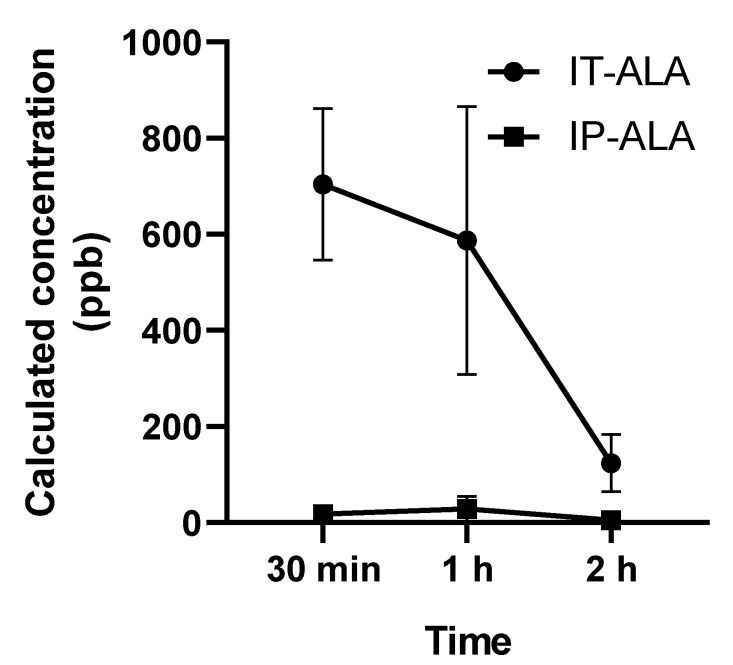
Perilymph concentration of ALA measured using UHPLC. The ALA concentration (parts per billion, ppb) in 3 μL of perilymph collected from the lateral semicircular canal demonstrated significantly higher concentration of ALA at 30 min and 1 h after the injection.

The ALA concentration (parts per billion, ppb) in 3 μL in perilymph collected from the lateral semicircular canal was measured using UHPLC at 0.5, 1, and 2 h after single drug administration. In the IT-ALA group, the ALA concentrations at 0.5, 1, and 2 h after injection were 703.9 ± 272.7 ppb, 586.7 ± 482.2 ppb, and 123.9 ± 2.3 ppb, respectively. In the IP-ALA group, the concentrations at 0.5, 1, and 2 h after injection were 17.6 ± 18.7 ppb, 28.7 ± 44.4 ppb, and 5.0 ± 2.3 ppb, respectively (Figure 3).

### 3.3. Morphometry of the OC

**Figure 4:**
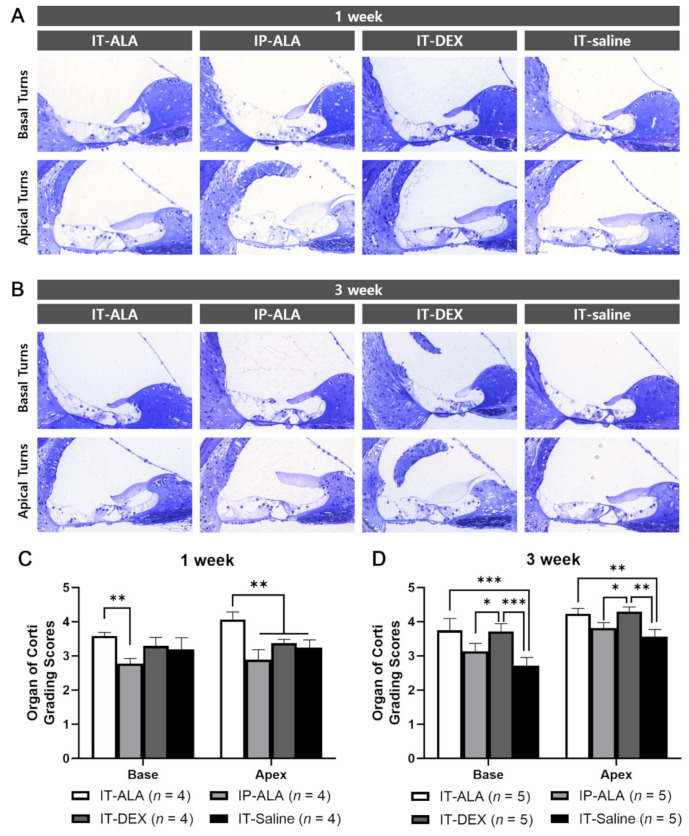
Morphological analysis of the OC in the mouse model of NIHL. Representative light microscopic findings at 1 week (**A**) and 3 weeks (**B**) after noise exposure. (**C**) OC grading scores were significantly higher in the IT-ALA group in the first week (*p* < 0.01). (**D**). IT-ALA and IT-DEX groups showed better OC recovery 3 weeks after noise exposure compared with IP-ALA and IT-saline groups (*p* < 0.05); however, significant difference was not observed between IT-ALA and IT-DEX groups (*p* > 0.05). OC, organ of Corti; IT, intratympanic; ALA, α-lipoic acid; DEX, dexamethasone; NIHL, noise-induced hearing loss. Mann–Whitney test with the Bonferroni corrections, * *p* < 0.05, ** *p* < 0.01, *** *p* < 0.001.

The OC morphologic score measured at one week after injection was significantly higher in the IT-ALA group (3.58 ± 0.20) than in the IP-ALA group (2.78 ± 0.25) in the basal turns (*p* = 0.005), and significantly higher in the IT-ALA group (4.06 ± 0.45) than in the other three groups in the apical turns (IP-ALA, 2.89 ± 0.51, *p* < 0.001; IT-DEX, 3.38 ± 0.21, *p* = 0.003; IT-saline, 2.72 ± 0.53, *p* = 0.002). At three weeks after injection, the IT-ALA group showed significantly better OC morphology compared with the IT-saline group in both basal (3.75 ± 0.77 vs. 2.72 ± 0.53, *p* < 0.001) and apical turns (4.23 ± 0.35 vs. 3.57 ± 0.47, *p* = 0.003), and the IT-DEX group showed better OC morphology than the IP-ALA and IT-saline groups (*p* < 0.05). Significant difference was not observed between the IT-ALA and IT-DEX groups in both basal and apical turns three weeks after drug administration (*p* > 0.05; Figure 4).

### 3.4. ABR Thresholds

**Figure 5:**
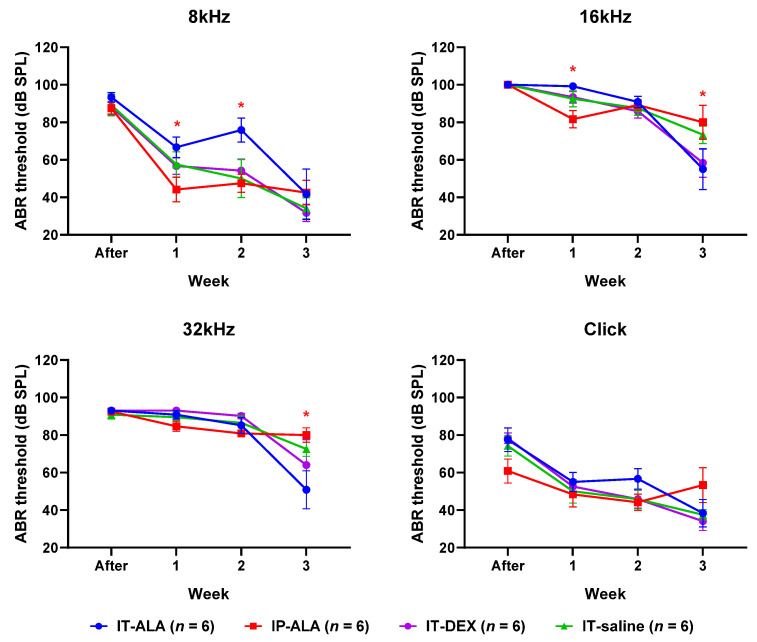
ABR thresholds. Mice in the IP-ALA group showed significantly better hearing than mice in the IT-ALA group at lower frequencies (8 kHz and 16 kHz) at 1 and 2 weeks after noise exposure. The IT-ALA group showed better hearing at 16 kHz and 32 kHz frequencies than the IP-ALA group 3 weeks after noise exposure. Significant difference was not observed between IT-ALA and IT-DEX groups. ABR, auditory brainstem response; IT, intratympanic; ALA, α-lipoic acid; DEX, dexamethasone. Mann–Whitney test with Bonferroni corrections, * *p* < 0.05.

The ABR threshold (Figure 5) in the IP-ALA group was significantly lower than in the IT-ALA group at 8 kHz (IP-ALA 34.2 ± 12.1 dB, IT-ALA 66.7 ± 14.7 dB, *p* = 0.01) and 16 kHz frequencies (IP-ALA 81.7 ± 10.8 dB, IT-ALA 99.2 ± 10.8 dB, *p* = 0.01) at one week after noise exposure. At two weeks after noise exposure, the ABR threshold was significantly lower in the IP-ALA group (35.75 ± 27.5 dB) than in the IT-ALA group (75.8 ± 27.3 dB) at the 16 kHz frequency (*p* = 0.003). However, the IT-ALA group showed a significantly lower ABR threshold at 16 kHz (IT-ALA 55.0 ± 25.6 dB, IP-ALA 80.0 ± 24.6 dB, *p* = 0.03) and 32 kHz (IT-ALA 51.7 ± 26.1 dB, IP-ALA 80.0 ± 27.5 dB, *p* = 0.04) three weeks after noise exposure. Significant difference was not observed between the IT-ALA and IT-DEX groups (*p* > 0.05). 

### 3.5. Immunofluorescence of Inflammatory Markers

**Figure 6:**
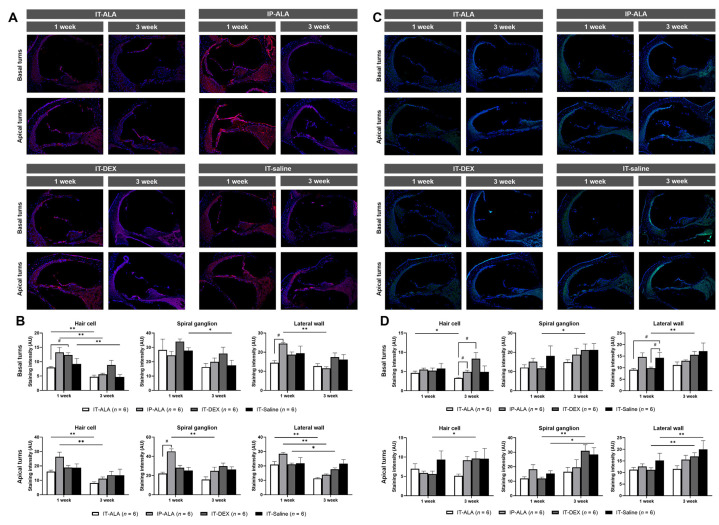
The expression of inflammatory markers in the mouse model of NIHL. A and B. Representative IL-1ß immunofluorescence findings (**A**) and quantitative analysis (**B**) showed IL-1ß expression was decreased in the IT-ALA group compared with the IP-ALA and IT-DEX groups 1 week after drug administration. Significant difference was not observed among the groups 3 weeks after drug administration. (**C**,**D**) Immunofluorescence results showed NF-κB expression was significantly decreased in the IT-ALA group. In addition, NF-κB expression was significantly increased in the IT-DEX and IT-saline groups 3 weeks after injection; however, the change was non-significant and even decreased in the IT-ALA group. IT, intratympanic; ALA, α-lipoic acid; DEX, dexamethasone; NIHL, noise-induced hearing loss.* Wilcoxon signed rank test, * *p* < 0.05, ** *p* < 0.01; # Mann–Whitney test with Bonferroni corrections, *p* < 0.05.

IL-1ß expression (Figure 6A,B) in the IT-ALA group was lower at one week after noise exposure compared with the IT-DEX group in the hair cells of basal turns (*p* = 0.02) and with the IP-ALA group in the lateral wall of basal turns (*p* = 0.03) as well as in the spiral ganglion of apex turns (*p* = 0.03). IL-1ß expression was higher in the IT-DEX and IT-saline groups three weeks after injection but without statistical significance. IL-1ß expression levels were decreased three weeks after one-week exposure to noise in all groups. In the IT-ALA group, IL-1ß expression significantly decreased in the hair cells in the basal turns (*p* = 0.006), hair cells of apical turns (*p* = 0.004), and lateral wall of apical turns (*p* = 0.004). In the IP-ALA group, the intensity decreased in the hair cells of basal turns (*p* = 0.004), lateral wall of basal turns (*p* = 0.004), hair cells of apical turns (*p* = 0.004), spiral ganglion of apical turns (*p* = 0.006), and lateral wall of apical turns (*p* = 0.004). In the IT-DEX group, decreased intensity was observed only at the lateral wall of apical turns (*p* = 0.04). In the IT-saline group, intensity decreased in the hair cells (*p* = 0.03) and spiral ganglion of basal turns (*p* = 0.04). 

NF-κB expression levels (Figure 6C,D) were significantly lower in the IT-ALA and IT-DEX groups one week after injection at the lateral wall of basal turns (*p* = 0.02 and 0.04, respectively). The NF-κB expression level was significantly increased at three weeks in the IP-ALA (hair cells of apical turns, *p* = 0.04), IT-DEX (spiral ganglion and lateral wall of both basal and apical turns, *p* < 0.05), and IT-saline (spiral ganglion and lateral wall of apical turns, *p* = 0.04) groups. However, significantly decreased NF-κB expression was observed in the hair cells of the basal turns in the IT-ALA group (*p* = 0.04). 

## 4. Discussion

The main findings of the present study can be summarized based on three perspectives: safety, efficacy, and mechanism. With regards to safety, IT-ALA injection induced temporary middle ear mucosal swelling that was recovered within three weeks. In terms of efficacy, IT-ALA injection showed a higher inner ear concentration compared with IP administration. IT-ALA showed a greater restorative effect on OC morphology and hearing compared with IP-ALA and demonstrated similar effects to IT-DEX injection after acoustic trauma. Regarding mechanism, IT-ALA injection apparently had more predominant downregulating effects on the inflammatory reaction based on IL-1ß and NF-κB expression in the cochlea compared with IP-ALA or IT-DEX injection after acoustic trauma. 

To confirm whether IT-ALA can be clinically applied to patients with acoustic trauma, safety evaluation was performed in the present study. To the best of our knowledge, this is the first study in which the safety of ALA in the middle ear was confirmed. Swelling of the submucosal layer was observed in the middle ear after IT-ALA injection but recovered within 3 weeks. In addition, the healing process of the tympanic membrane was similar to IT-DEX injection. Therefore, IT-ALA injection is expected to be safe in the middle ear concerning inflammation. Regarding the inner ear safety of ALA, in a previous study in which HEI-OC1 cells were used, a decrease in cell viability at an ALA concentration of 500 μM (approximately 103,165 ppb) or higher was reported [30]. Therefore, the ALA concentration measured at the perilymph after IT-ALA injection in the present study (703.9 ± 272.7 ppb) does not appear to be cytotoxic.

In the present study, IT injection ensured higher inner ear concentrations of ALA than IP administration, which has also been confirmed in a previous pharmacokinetic study in which administration methods were compared using guinea pigs [16]. In addition, the functional efficacy of IT-ALA injection based on the results of hearing tests was demonstrated in the present study. In previous studies, ALA showed oto-protective effects in several animal models of drug-induced ototoxicity [11,27], acoustic trauma [22], age-related hearing loss [12], and hyperglycemia-related hearing loss [31]. However, in most of the studies to date, the focus was on the protective effect of ALA rather than its rescue effect. Because oxidative damage is considered a cause of acute inner ear diseases such as ISSNHL and NIHL [29,32], studies in which antioxidants are used for hearing rescue are needed. Therefore, the present study was conducted, and results showed IT administration of ALA was safe and had a better hearing rescue effect with higher concentration of the drug in the cochlea com-pared with IP administration.

Various action mechanisms of the two drugs may explain the different expression levels of the inflammatory markers within the cochlea between the IT-ALA and IT-DEX groups. IL-1ß and NF-κB are inflammatory markers activated due to ROS stimulation induced by acoustic trauma [29]. In a previous study, a biphasic elevation of IL-1ß was reported after acute acoustic trauma: the first peak at 6 h and the second peak at 7 days after noise exposure [33]. In another study, a significant increase in both IL-1ß and NF-κB expression was observed until seven days after acoustic trauma that was reduced by another antioxidant (Q-ter) and anti-inflammatory (IL-1 receptor antagonist, anakinra) treatment [29]. In the present study, IL-1ß and NF-κB expression were evaluated in the cochlea at one and three weeks after noise exposure to investigate the inflammatory response at one week and later. Steroids are effective drugs for acute hearing loss; however, the action mechanism remains poorly understood [34]. In a previous study, DEX could not inhibit inflammatory gene expression (including IL-1ß) but increased the expression of ion homeostasis-related genes [35]. Similarly, mice in the IT-DEX group did not show suppressed but conversely increased IL-1ß expression at one week after administration in the present study. Proinflammatory cytokines, including IL-1ß, are thought to cause the apoptosis of hair cells via influx of inflammatory cells [33]. The results of previous studies and the present study indicate the effect of DEX on restoring the inner ear condition after acoustic trauma may not correlate with the inhibitory effect of inflammation associated with IL-1ß and NF-κB. In another study that included patients with autoimmune inner ear disease, a higher IL-1ß level was observed in peripheral blood samples of corticosteroid non-responders [36]. Conversely, in the present study, ALA significantly decreased the expression levels of IL-1ß and NF-κB in the cochlea with restored hearing after acoustic trauma. 

Our study had several limitations. Because the main purpose of this study was to investigate the safety and efficacy of IT-ALA, the precise mechanism-related studies of ALA in the inner ear were not conducted. In order to elucidate the mechanism of action of IT-ALA in the inner ear, extensive analysis, including inflammasome, pro-inflammatory, and ROS markers will be required in the further studies.

However, we propose ALA can be one of the rescue drugs for acoustic trauma. For patients who do not obtain satisfactory therapeutic effects from DEX alone, supplementary treatment with IT-ALA could be considered based on the results of this study, although further clinical studies need to be performed before its clinical application.

## 5. Conclusions

In the present study, IT injection of ALA was shown to be safe. Both histologic and audiologic results demonstrated a better rescue effect after acoustic trauma with a higher perilymphatic concentration of the drug in the IT-ALA group than in the IP-ALA group. In addition, the efficacy of IT-ALA injection after acoustic trauma was similar to IT-DEX injection although their therapeutic action mechanism appears to be different.

## Data Availability

The data is contained within the article.
